# Schizophrenia in a member of the family: Burden, expressed emotion and addressing the needs of the whole family

**DOI:** 10.4102/sajpsychiatry.v22i1.922

**Published:** 2016-08-31

**Authors:** Gian Lippi

**Affiliations:** 1 Department of Psychiatry, University of Pretoria, South Africa

## Abstract

How often do we find ourselves concentrating so much on treating a patient with schizophrenia that we forget about the needs and difficulties of the family members who take care of that patient? This article highlights the global and specific difficulties that families and caregivers experience in having to care for chronically ill family members with schizophrenia with a backdrop of continuing global deinstitutionalisation of such patients. Matters such as burden and expressed emotion are explored, family-specific interventions are discussed and areas of service delivery and resource inadequacies are identified.

## Introduction

Schizophrenia is a psychiatric disorder, which is characterised by slow functional deterioration and episodes of relapse or acute exacerbation of psychotic symptoms. The mean age of onset in early adulthood, deterioration in patients’ activities of daily living and ability to sustain employment, and the propensity of the disorder to affect insight leave many patients requiring assistance and care for an extended period of time.

At the same time, the global push for the deinstitutionalisation of these patients has resulted in an increase in responsibility for care to be supplied by the family and its members.^1,2,3,4,5,6,7^ Unfortunately, there is also evidence worldwide that this policy of deinstitutionalisation, and the rate thereof, has not been matched by a sufficient increase in community mental health resources everywhere, that is, resources that can assist both patient and family. This is especially true in developing countries where insufficient financing severely restricts the development of these community resources. In essence, governments worldwide are entrusting the long-term care of their patients with schizophrenia to family members. The question is, ‘how much do these family members know about the disorder or how to manage people who suffer from it?’ Are they equipped for the task? The literature suggests that they are uninformed and ill-equipped.^6,8^ In this context, studying the impact of caregiving on these families therefore becomes especially relevant.^9^

Caregivers of patients with childhood onset chronic psychiatric disorders such as autism spectrum disorders, who are usually the parents, realise at an early stage that there will be a responsibility for them to care for their child for the rest of their lives in most cases. They therefore tend to adapt accordingly as the child grows up and experience a comparatively slow change to their lives and expectations regarding their ill child.

On the other hand, major neurocognitive disorders tend to affect the elderly. There is therefore a sudden responsibility thrust upon carers (in this case, mostly spouses or children) that requires a dramatic adjustment to their lives; however, because of the life expectancy associated with illnesses that present with the disorder, it is a comparatively short-term responsibility.

Patients with schizophrenia can often have a normal childhood and adolescence before suddenly, unexpectedly and often dramatically becoming ill. Because of the age of onset, care responsibilities are suddenly thrust upon mostly parents, even before they have come to terms with the shock of the sudden, dramatic onset of the illness. It often comes at a time when they would expect their child to gain independence and when they themselves are at an age when retirement could have been considered. The lowering of expectation for the future of their child, along with the new, long-term care responsibilities, tends to weigh heavily on these parents, requiring a dramatic adjustment to their lives and subjecting them to unique symptoms and behaviours, which become increasingly difficult to manage, especially for people of their age. Resultant negative effects on the family are not surprising.

There has been research into the problems these families face, especially in the context of continuing deinstitutionalisation. Before these families can be helped, one needs to first identify and understand the problems they experience.

## The concept of burden

Caring for family members with schizophrenia subjects caregivers to mostly negative experiences, which in turn negatively impact the caregivers themselves. Table 1, ‘second column, provides information and examples of how caring for family members with schizophrenia can negatively impact the caregivers themselves. These negative aspects experienced by patients’ relatives as a consequence of their caregiving role are collectively known as ‘burden’.^10^ Attempts have been made in the literature to better define ‘burden’ as the existence of serious psychosocial and emotional problems, difficulties or negative events,^4^ stressful situations or significant life changes that influence the family member of an ill relative.^11^ It has also been defined as the extent to which caregivers perceive their emotional and physical health, social life and financial status as suffering as a result of caring for a relative.^12^

**TABLE 1:** Examples of objective and subjective burden as experienced, and described, by family members or caregivers.

Burdens	Description by family members and caregivers
Objective Burdens	Neglect of other family members and disruption of family life, ^9,12,18,20,21^ deterioration in social and family relationships and matrimonial problems/breakdown.^3,6,12,13,18^ Disruption and constraints in daily social,^3,10,21-23^ work^10,18^ and leisure activi-ties.^3,6,10,18,20,21,23,24^ Social isolation and lack of social support.^18,25^ Withdrawal of support by/loss of contact with friends, family and neighbours.^6,13^ Loss of employment/income or reduced productivity/increased absenteeism.^18,23^ Increased medical expenses and financial problems.^6,10,12,13,16,18,20,22,23^ Increased workload and taking over of tasks like shopping, repairs, clothes washing and minor chores.^11^ Changes to household routines.^6^ Neglect of hobbies.^10,21^ Difficulties in going on holidays/Sunday outings.^10,18,21^ Difficulties in inviting people to one’s home.^10^ Supervisory obligations and having to accompany the patient outside the home.^13,18^ Having a chaotic lifestyle and poor quality of life.^2,3,12,17,18,24,25^ Need for care services.^16^ Experiencing stigma related to the illness.^18,23^
Subjective burdens	Guilt and self-blame,^6,18,22,26^ for not recognising symptoms earlier and/or for being the cause of the illness (‘schizophrenic mother theory’).^16,19,27^ Apathy and denial of illness.^3,28^ Feelings of loss (of the potential of the family member).^3,10,19,22,27^ Worry - mostly about the patient’s future.^3,6,17,18,22^ Fear (of violence especially).^3,22^ Tension,^17^ anxiety,^5,6,10,22,24,25^ ‘stress’ and ‘shock’.^3,22^ Dejection, grief,^3,19,22^ sadness,^3,21^ crying and distress – leading to depression.^3,5,6,10,21,22,24,25,29^ Emotional costs/wellbeing,^12,15^ mental health problems and psychological morbidity.^17,18,23,29^ Physical problems.^18,27^ Feelings of resignation,^16^ resentment, confusion,^22^ loss of control,^3^ desperation and frustration.^29^ Helplessness and hopelessness.^3,27^ Aloneness and emptiness.^29^ Embarrassment in social situations,^10,16,18^ humiliation and shame.^3,6,18,27^ Feelings of having no influence on the illness despite self-sacrificing care.^16^ Feelings of being incapable of caring adequately for the patient.^3^ Exhaustion from increased energy expended on dealing with problematic patient behaviour, psychotic symptoms, poor self-care, reclusiveness, poor medication adherence and confusion.^3,27^ Lack of sleep created by excessive noise from the patient.^13^ Emotional effort expended in encouraging patient activity and medication adherence.^13^ Low self-esteem and feelings of inferiority.^3,22^ ‘at wit’s end’, ‘feeling marginalized’ and ‘lacking support’.^3,29^

Burden can be classified according to the affected party. For example, family burden is the burden experienced by the family as a unit,^12,13^ while individual burden is experienced by a single caregiver or individual member of a family.

Burden is not only the objective demands associated with caring but also the caregivers’ subjective reaction to them.^14^ Reference is therefore made to objective burden being the practical problems, difficulties and concrete and observable negative effects the illness has on family life resulting in significant life changes.^10,12,15,16,17,18^ On the other hand, subjective burden is the extent to which caregivers actually feel burdened because of the situation,^16^ resulting in psychological reactions and affecting well-being.^10,12,15,16,17,18^

For examples of objective and subjective burden experienced, and described, by family members or caregivers, see Table 1.

Objective and subjective burdens have a direct influence on each other. For example, decreased participation in social activities as a result of having to spend many hours caring for an ill relative can lead to increased depressive symptoms, which, in turn, can lead to a decreased drive to participate in social activities.

Research has revealed that family members caring for relatives with schizophrenia experienced significantly higher levels of objective and subjective burden than those caring for relatives with chronic physical illnesses or other chronic psychiatric disorders such as depressive disorders, bipolar disorders and obsessive–compulsive disorder.^4,10,17^ Severe objective and subjective burdens increase the global burden experienced. Parents of patients with severe and permanent psychosocial functional impairments have been shown to have a constantly high level of global burden.^16^

Many factors have been identified which can either increase or decrease the severity of burden experienced (Table 2).

**TABLE 2:** Factors influencing burden.

Burdens	Factors
Increased Burdens	Severely ill/disabled patient.^11,17,21,28^ Unemployment/low level of patient psychosocial functioning of.^11,12,15,17,21,23^ Patient presenting with severe negative symptoms and poor self-care.^12,22,31^ Acutely psychotic patient,^16^ severe positive symptoms and undue suspiciousness.^12,17,22^ Patient with concomitant symptoms like obsessions and phobias.^22^ Patient hostility,^21^ aggression,^16,22^ disruptive symptoms,^31^ violence,^11,22^ property damage,^22^ childish/sexually inappropriate behaviour,^16,22^ rapid mood changes and behavioural disturbance.^22^ Patient suicidal ideation/self-harm.^31,22^ Patient sleep-wake cycle disturbance.^22^ Initial onset and early stages of patient illness.^11,12,14,17,31^ Patient symptom relapse/deterioration.^16^ Patient hospitalization.^14^ Frequent hospital visits.^17,31^ Poor patient medication adherence.^15^ Young/old or male patient.^17,28,31^ Low level of patient and caregiver education.^12,17,28^ Female and/or unmarried caregiver.^16,17^ Young/old caregiver age.^4,12,17,26,28^ Parents/close relatives as caregivers.^4,12^ Passive caregiver coping skills (eg. avoid-ance/denial).^14,17,28,31^ Poor caregiver quality of life.^12,17^ Large number of hours spent caring for the patient (> 7hours daily – ‘role overload’).^11,12,15,17,32^ Limited friend/family care involvement.^4^ Non-nuclear family/negative family atmosphere with frequent arguments (decreased sense of coherence).^4^ Poor family resources,^31^ greater family demands and lower income/socio-economic background.^4,12,17^ Family home far from hospital/in an urban setting.^17^ Experiencing illness-related stigma.^22^
Decreases burden	Improved patient condition.^16^ Long periods of remission.^12,16^ Good relationship with patient/mutuality.^4,33^ Patient participation in a rehabilitation programme.^15,23^ Family participation in a psycho-education programme, group therapy/in a self-help group.^23,25,29^ Home visits by nurses as part of an individualized care plan.^23^ High social support.^12,14,31^ Professional network support.^21^ Caregiver optimism.^33^ High active caregiver coping mechanisms.^14,31^ Caregiver religious coping strategies.^28^ Male caregiver.^28^ Sibling/close relative/friend as caregiver.^4^ Caregiver problem-solving skills.^2^

Caregivers rarely voice the burden experienced,^19^ resulting in professionals often not being fully aware of the negative impact of caring for a mentally ill relative on caregivers. As this burden has now been intensively researched and is better understood, awareness will increase and these family members should receive the help they deserve in an attempt to decrease the burden experienced.

## The influence of expressed emotion on schizophrenia

Expressed emotion (EE) can be interpreted as a complex pattern of interaction between the patient and his or her family that at the same time represents the general conditions and consequences of the mental illness.^16^

It changes over time and is influenced by circumstance – it decreases after a patient is discharged from hospital and then slowly increases again.^33^ EE is measured through five variables that reflect carer attitude: hostility, critical comments, positive comments, emotional over involvement (EOI) and warmth.^34^

These variables collectively, but not individually, influence relapse rates.^29^ Families that tend to be hostile, critical and emotionally overinvolved are said to be high expressed emotion (HEE) families,^3,19,29,35^ while families that tend to be positive, empathetic, calm and respectful, with low levels of emotion, are said to be low expressed emotion (LEE) families.^19^ Families with HEE tend to believe that the symptoms can somehow be controlled by the patient^35^

Warmth, previously seen as an attribute of LEE, has been found to be an unreliable variable of EE because high levels of warmth (which is positive) are accompanied by EOI, while low levels of warmth are accompanied by an increase in critical comments – EOI and criticism both being variables in HEE (which is negative).^35^

EE is a transcultural phenomenon and a very reliable predictor of relapse in schizophrenia.^3^ HEE is the third most common cause of relapse behind non-adherence with medication and drug abuse with 50% of patients discharged back to HEE families soon relapsing compared to a 21% relapse rate after discharge back to an LEE family.^35^

Another negative finding is that levels of EE are highly resistant to change, with short educational interventions having no influence on levels of EE or EOI.^6^ HEE is especially resistant, with intensive interventions required to decrease levels of EE, even then it is often unsuccessful. Recent evidence does, however, indicate that although levels of EE can temporarily decrease with intervention, the levels return to the prior levels of HEE within 3 years, probably coinciding with a decrease in the level of functioning of the patient. There is contradictory opinion as to whether interventions have any effect on EE.^3,33^ However, proper family psycho-education not only on the illness but also on the subject of EE is essential. Advice on the need to discontinue practices that lead to HEE and EOI – such as criticism of the patient (for instance, for ‘being lazy and sitting around doing nothing’ when in fact he/she experiences negative symptoms of schizophrenia) and intrusiveness (through invasion of the patient’s privacy and emotional and symptomatic probing) – should be given to families.

## Inadequacies in resources and service delivery

Mental health care resources and levels of service delivery vary greatly worldwide; yet there are definite similarities in the experiences of family members of mentally ill patients dependent on mental health care service. Negative experiences and complaints are in all likelihood highly subjective and generalised. However, whether or not these complaints are fair is another point of discussion. They merit our attention if we wish to succeed in our quest of helping these families. Table 3 presents common complaints about resources, service delivery and mental health care professionals.

**TABLE 3:** Common complaints by families and caregivers about resources, service delivery and mental health care professionals.

Common Complaints	Comments
Complaints about resources and service delivery	That there is a: – general poor level of mental health care;^27^ – lack of support from social psychiatric services;^5,19,27^ – lack of help centres, crisis support facilities and self-help groups (most of which are privately funded by committed relatives);^27^ – refusal of health insurance to cover therapeutic sessions;^27^ – lack of support from the State and from health insurance regarding financial burden;^27^ – problem surrounding laws, legislation, regulations, politics and bureaucracy within mental health care;^27^ – lack of co-ordination and allocation of responsibility within mental health care.^27^
Complaints about mental health care professionals	Professionals: – show a lack of interest in the patient, as well as the family and their fears, problems and worries;^27^ – seem uncaring and disrespecting;^3,29^ – are believed to regard family members as a burden and a source of irritation;^27^ – are experienced as being arrogant;^27^ – are believed to exclude family members from the treatment pro-cess;^27^ – use technical language, seemingly as a means of excluding rela-tives;^27^ – offer no hope and don’t validate family member concerns/give simple explanations;^19^ – disregard family members’ knowledge of the patient through failure to consult with them/seek their opinion;^19^ – make it difficult for family members to get relevant information from them;^22^ – withhold diagnoses;^22^ – are seen to discriminate against families, who believe that their family member with a mental health problem does not receive the same level of care as patients with physical problems.^29^ There is a lack of: – support from professionals,^25,29^ especially psychologists, social workers and psychiatrists;^22^ – engagement from staff.^25^

Most families of patients with schizophrenia believe that they received inadequate information regarding the illness of their relative,^19,20,21,25,27,36^ early warning signs of relapse, effects of medication and ways of coping with bizarre and violent behaviour.^8^

Another complaint is that the police do not know enough about mental illness, are badly trained and make fun of patients.^27^

It is a poor reflection of service delivery when the families believe that they receive more support and information about mental illness from self-help groups than from mental health care professionals.^22^ It has also been proved that the lack of community mental health resource centres and perceived support from mental health care departments increase levels of burden experienced by families of patients with schizophrenia.^19^ In most areas of South Africa, there is currently a distinct lack of community resources to deliver services that can contribute positively to the lives of these families.

## Concepts for addressing the needs of these families

There are difficulties experienced by patients with schizophrenia and caregivers and families which are shared, but there are also difficulties which are unique to each. The difficulties are complex, and it is suggested that they should be addressed both individually where appropriate and together where possible. One can thereby categorise the focus of service delivery into the following sections:

1. Helping the patients
2. Helping the families to help the patients
3. Helping both the patients and the families
4. Helping the families.

### Helping the patients

As discussed above, by increasing the levels of psychosocial functioning and decreasing hospitalisations, psychotic and negative symptoms of the patient, levels of family burden are also decreased. Optimal treatment focussed on minimizing and controlling symptoms by optimizing medication should be strived for. Psychosocial and psychotherapeutic interventions directed at improving insight and levels of functioning are equally important. Thus, by helping the patients, the families are helped indirectly.^13^

### Helping the families to help the patients

Families require advice surrounding how best to care for their ill relative and positively contribute towards maintaining his or her good mental health. Providing families with sufficient information about the disorder,^10,37^ early symptoms and signs of relapse and side-effects of prescribed medication, as well as ways to deal with bizarre and violent behaviour and various strategies for patient management at home is therefore essential.^37^ One of the most important contributions that families can make to the well-being of their ill relative is to lower the levels of EE within the family, the concept of which is discussed above. Families require detailed information about EE and how it influences the mental state of patients with schizophrenia so that they can make necessary adjustments and positively influence the mental state of their relative. Interventions aimed at helping these family members directly can also assist them with helping the patient.

### Helping both the patients and the families

Successful treatment of the patients and implementing specific interventions for both patients and families go a long way in benefiting both parties. A lot of what can be done to assist both patients and families are practical in nature and involve resources that are not only visible but also tangible in other ways. In the literature, there are practical means of helping both patients and families (see Box 1). Some of the resources mentioned do exist in certain areas locally (but small in numbers), but are distinctly lacking in other areas. Other elements from Box 1 may exist but are not necessarily implemented as optimally as would have been originally envisioned.

BOX 1 Practical ways of helping both the patients and the families.

- Creation of more mental health care resource centres and support facilities.
- Creation of more support and self-help groups.^27^
- Creation of long-term residential facilities with different levels of supervision of these patients.
- Creation of home treatment, assertive outreach and crisis and early intervention teams consisting of different mental health care professionals.^20^
- Creation of halfway-houses, day hospitals/similar rehabilitation centres to provide overburdened caregivers with much needed respite.^38^
- Creation of more job opportunities for these patients and making work rehabilitation a priority, whilst stressing the importance of occupational therapy.^27^
- Creation of complaint services.^27^
- Giving out essential information about legal regulations, housing and financial support and brochures containing telephone numbers and addresses of where help can be found.^27^
- Education and training, like seminars for professionals, relatives and lay persons.^27^
- Inviting journalists to self-help groups and promoting a positive image of mental illness in the media in order to reduce stigma and discrimination.^27^
- Improved mental health care legislation.^27^
- Improved communication between professional and relatives.^27^
- Supervision of psychiatric institutions by an independent body.^27^

### Helping the families

In order to help the family members directly, they have to be seen as index patients themselves and be offered therapies and interventions aimed directly at improving their well-being when necessary. Addressing their worries, fears and symptoms of depression and anxiety, which develop as a result of caring for mentally ill relatives, may require situation-specific therapeutic input. Providing family member caregivers with higher levels of support, attempting to increase their levels of optimism and including them in the treatment process of their ill relatives are vital elements that need attention in order to positively influence the long-term mental health of these people.

Specific interventions and programmes aimed at addressing the needs of families and empowering them with relevant information and skills have been well researched and implemented in selected areas worldwide. Some of these are outlined in the next section.

## Interventions and programmes

Apart from specific rehabilitation programmes for patients, which result in fewer health and economic problems and decreased family burden and disruption of social lives of caregivers,^15^ there are examples of interventions and programmes that consist of different combinations of strategies aimed at family members of patients with schizophrenia. There is evidence of their efficacy, but they are not always being readily implemented.^10,20,25^

Interventions and programmes are built around psycho-education (PE) and cognitive behavioural therapy (CBT) and involve elements such as problem-solving and coping strategies.^10,28^ Religious coping is an interesting form of coping that can be implemented in interventions as it has been shown to decrease burden. Believers indicate that feeling that a ‘Creator’ was in control decreased the level of distress and increased their hope for the future.^28^ Interventions need to be implemented early; otherwise, relatives will develop fixed views that might be difficult to change.^6^

PE is focussed on providing patients and families with information about early signs of relapse and effects of medication. Research indicates that families tend to not find the information too complicated to understand.^22^

CBT is aimed at increasing optimism. Some programmes with the most robust evidence for efficacy are outlined below.

### Psychoeducational interventions

- Various models exist, including the Support and Family Education Programme.^36^ Not all are diagnosis specific.
- Aims:
  1. To reduce relapse rates by providing skills training in problem-solving, communication and coping skills^25^
  2. To share information about the disorder, its course, early warning signs and relapse prevention^25,36^
  3. To give families opportunities to ask questions about psychiatric disorders and treatment options^36^
  4. To help families understand the importance of early intervention^36^
  5. To change the families’ level of EE^6^
  6. To publicise the availability of mental health services^36^
  7. To reduce the stigma of mental illness.^36^

- Group interventions consist of the following:
  1. Provision of new information – presentations are given and families are provided with newsletters, pamphlets, lists of resources and websites, and may borrow books and videos (relatives tend to find videos the most informative and presentations more informative than the literature).^6,25,36^
  2. Group discussions and sharing of experiences.^6,25,36^
  3. Question and answer sessions.^25,36^

- Individual family sessions are also provided and they consist of behavioural family therapy (BFT).
- The programmes usually consist of 12 or more sessions of 90 minutes each over 6 months to 2 years.^25,36^
- Results:
  1. Increased knowledge about schizophrenia^26^
  2. Increased satisfaction with health care services^6^
  3. Increased use of coping behaviours^6^
  4. Increased optimism^6^
  5. Reduced anxiety, stress and distress^6^
  6. Improved patient medication adherence^38^
  7. Reduced patient relapse and admission rates^25,38^
  8. Reduced family burden^13,25,29^
  9. No change in levels of EE or EOI.^6^

### Peer-led family support and psycho-education

- An example of a programme with an evidence base for efficacy from randomised controlled trials is the National Alliance on Mental Illness’s Family-to-Family Program.^7^
- The aims of this programme and other similar programmes are to help families get information, access support and services, improve coping skills, engage in self-care, improve communication, increase empathy, solve problems and understand research that promotes recovery.^7^
- The concept surrounds the utilising of individuals who have experience living with illnesses such as schizophrenia for coaching, mentoring, teaching, coping and advocacy guidance (a model not dissimilar to that of substance abuse recovery).^7^
- It consists of the following:
  1. A 12-week skill-building course taught by family members (who receive specific training to lead and facilitate sessions) for family members.^7^
  2. Sessions of two and a half hours each (up to 14 sessions).^38^

- Results:
  1. Improvements in knowledge and both emotional and problem-focussed coping^7^
  2. Improved problem-solving and reduced distress and subjective burden^25,38^
  3. Improved family coping^38^
  4. Improved overall functioning of the patient (including self-maintenance, social functioning and community living skills) and decreased number and durations of hospitalisations.^38^

### Behavioural family therapy

- It is a family psycho-educative intervention which addresses stress management and goal achievement.^34^
- Positive results include decreased family burden, relapses and dosages of neuroleptics taken by patients. It also decreases feelings of resignation and increases optimism in caring for the patient. Families gain coping strategies and independence.^34^

### Multiple family group therapy

- It is a combination of BFT and formal PE which also concentrates on problem-solving.^14,30^
- It aims to have a positive effect on patient outcome by decreasing symptoms and relapse rates and increasing social and vocational skills.^14,30^
- The group consists of two clinicians and eight families.^14,30^
- The programme consists of four phases as follows:
  1. the joining phase
  2. a 1-day PE workshop
  3. a year of fortnightly sessions focussing on relapse prevention
  4. a year of monthly sessions focussing on social and vocational rehabilitation.^14,30^

- Results include reduced negative symptoms and hospitalisations, but it is not primarily aimed at or proven to decrease burden.^14,30^

### Family interventions

- These interventions explore solutions to help families effectively deal with difficulties that occur secondary to illness, and promote understanding of psychosis with the goal of improving patient social functioning and independence.^20,34^
- Aims:
  1. To instil hope by focussing on recovery^20,34^
  2. To provide education and information^20,34^
  3. To implement strategies to reduce stress and distress^20,34^
  4. To enhance existing coping strategies^20,34^
  5. To foster effective communication^20,34^
  6. To promote independence^20,34^
  7. To develop a staying well plan^20,34^
  8. To reduce HEE^33^
  9. To prevent psychotic relapse.^33^

- It consists of:
  1. an education session^20,34^
  2. problem-solving sessions using CBT^20,34^
  3. sessions concentrating on practical ways of dealing with emotion.^20,34^

- The programmes contain elements of stress management, communication skills, problem-solving and goal achievement and improve knowledge about schizophrenia and the early warning signs^20,34^
- Results:
  1. An increase in the understanding of the illnesses^38^
  2. Decreased family tension and stress^20,34^
  3. Decreased burden^20,34^
  4. Decreased EOI^33^
  5. Decreased rates of relapse and hospitalisation^3,38^
  6. Improved medication adherence.^38^

Barriers to implementing these interventions in practice are time, funding and the availability of supervision.^20^ Just because the likelihood of local government and mental health care services being able to create enough resources to implement these interventions and programmes is not that realistic does not mean that mental health care professionals in this country are unable to help families of patients with schizophrenia. Most existing mental health care facilities have resources to supply educational interventions. Brief educational intervention in the setting of a consultation can greatly reduce subjective burden in family members of mentally ill patients.^6^ A feeling of support from mental health care services can be created within caregivers of mentally ill patients by using simple phrases of encouragement such as ’hang in there’, ‘never give up’ and ‘take it one day at a time’.^19^ Furthermore, giving comparatively insignificant pieces of advice can dramatically decrease levels of family burden. For example, in families where there is more than one caregiver, they should take turns in caring for the patient and should make full use of their time away from the patient by maximising time for hobbies and leisure. Advice should be given that they should structure time to just ‘get away from it all’ and ‘let their hair down’.

## Conclusion

Families of patients with schizophrenia experience high levels of burden and receive very little information about the illness, how to cope with their mentally ill relative or related matters such as EE. They are subsequently rendered largely ill-equipped to deal with these challenges and problems. By providing more information and resources, and implementing programmes designed to address these problems, families of patients with schizophrenia can experience higher levels of support and empowerment and lower levels of burden. Furthermore, families need to be psycho-educated about the negative effects of patient criticism and intrusive behaviours which increase levels of HEE and EOI, both of which are detrimental to patients and increase relapse rates.
